# Predictive Performance of a Seven-Plex Antibody Array in Prenatal Screening for Down Syndrome

**DOI:** 10.1155/2015/519851

**Published:** 2015-04-23

**Authors:** Jeroen L. A. Pennings, Sandra Imholz, Ilse Zutt, Maria P. H. Koster, Jacqueline E. Siljee, Annemieke de Vries, Peter C. J. I. Schielen, Wendy Rodenburg

**Affiliations:** ^1^Centre for Health Protection (GZB), National Institute for Public Health and the Environment (RIVM), P.O. Box 1, 3720 BA Bilthoven, Netherlands; ^2^Centre for Infectious Diseases Research, Diagnostics and Screening (IDS), National Institute for Public Health and the Environment (RIVM), P.O. Box 1, 3720 BA Bilthoven, Netherlands; ^3^Department of Obstetrics, Wilhelmina Children's Hospital, University Medical Centre Utrecht (UMCU), P.O. Box 85090, 3508 AB Utrecht, Netherlands

## Abstract

We evaluated the use of multiplex antibody array methodology for simultaneous measurement of serum protein markers for first trimester screening of Down Syndrome (DS) and other pregnancy outcomes such as preeclampsia. For this purpose, we constructed an antibody array for indirect (“sandwich”) measurement of seven serum proteins: pregnancy-associated plasma protein-A (PAPP-A), free beta subunit of human chorionic gonadotropin (f*β*-hCG), alpha-fetoprotein (AFP), angiopoietin-like 3 (ANGPTL3), epidermal growth factor (EGF), insulin-like growth factor 2 (IGFII), and superoxide dismutase 1 (SOD1). This array was tested using 170 DS cases and 510 matched controls drawn during the 8th–13th weeks of pregnancy. Data were used for prediction modelling and compared to previously obtained AutoDELFIA immunoassay data for PAPP-A and f*β*-hCG. PAPP-A and f*β*-hCG serum concentrations obtained using antibody arrays were highly correlated with AutoDELFIA data. Moreover, DS prediction modeling using (log-MoMmed) antibody array and AutoDELFIA data gave comparable results. Of the other markers, AFP and IGFII showed significant changes in concentration, although adding these markers to a prediction model based on prior risk, PAPP-A and f*β*-hCG did not improve the predictive performance. We conclude that implementation of antibody arrays in a prenatal screening setting is feasible but will require additional first trimester screening markers.

## 1. Introduction

Since the introduction of prenatal screening for Down Syndrome (DS), much international research effort has been put in improving the predictive performance of prenatal screening for DS and other aneuploidies, by identifying novel serum protein biomarkers and, since more recently, the use of plasma cell-free DNA (cfDNA). Additionally, several studies have looked into serum proteins as potential biomarkers for other pregnancy outcomes, especially for preeclampsia (PE) and intrauterine growth restriction (IUGR). The underlying goal of many of these studies was to combine multiple biomarkers into a prenatal screening for DS and/or other pregnancy outcomes.

Whereas current serum screening methods require additional sample volume for each additional marker, other immunoassay methods have emerged that allow measuring multiple biomarkers in a constant and relatively small volume of sample material. More specifically, an antibody array (Ab-array) provides a high-throughput platform for protein expression profiling, allowing simultaneous measurement of several dozens of markers, while only requiring fingerprick amounts of blood [1]. Ab-arrays have therefore considerable potential to help increase the performance in first trimester screening for DS, as well as other pregnancy outcomes.

In previous studies, we identified candidate DS screening serum markers by human serum analysis using multiplexed immunoassays [2] and data mining using literature resources [3] and by the use of a mouse model for DS [4]. Additionally, we identified candidate markers for PE by data mining [5] and serum analysis. In parallel to biomarker identification, we developed and optimized a duplex antibody array for the two serum markers used in the first trimester combined test for DS, namely, pregnancy-associated plasma protein-A (PAPP-A) and the free beta subunit of human chorionic gonadotropin (f*β*-hCG). Serum samples from pregnant women, representing the dynamic range of both markers, were analyzed on these Ab-arrays, and the results validated to the results obtained by the, in prenatal screening routinely applied, AutoDELFIA system [6]. In a subsequent study, we showed that this Ab-array can also be used for serum collected by finger prick, as well as blood samples obtained by dried blood spot (DBS).

The aim of this study was to evaluate the use of a multimarker Ab-array in a prenatal screening setting. For this purpose, we expanded our previously developed Ab-array with five candidate DS and/or PE markers identified in our previous studies as suitable candidates. This marker set was composed to represent the multiple approaches used to identify candidate markers. For each approach, we selected those markers that had a high strength of evidence in one or more of our previous studies, for which commercially available antibodies could be obtained. The five selected markers were alpha-fetoprotein (AFP), angiopoietin-like 3 (ANGPTL3), epidermal growth factor (EGF), insulin-like growth factor 2 (IGFII), and superoxide dismutase 1 (SOD1). The resulting multiplex Ab-array was subsequently applied for a large-scale evaluation study using 680 serum samples obtained as part of the national DS screening program. The study comprised the evaluation of Ab-array serum analysis methodology, individual marker performance, and multimarker based risk prediction models.

## 2. Methods

### 2.1. Serum Samples

Sera of 170 DS pregnancies and 510 unaffected control pregnancies were retrieved from −80°C storage at the serum bank of the Dutch National Institute for Public Health and the Environment (RIVM). These samples were collected as part of the routine first trimester Down Syndrome screening program. Each DS sample was matched to three control samples by gestational age (exact day where possible, otherwise ±2 days), maternal weight (±10 kg), and maternal age (±5 year) and by closest sample date to obtain a single control group of sufficient size for statistical calculations. All blood samples were drawn during the 8th–13th weeks of pregnancy (between 58 and 96 days of gestational age). As part of the first trimester screening, PAPP-A and f*β*-hCG concentrations were previously measured with an automated dissociation-enhanced lanthanide fluorescent immunoassay (AutoDELFIA; PerkinElmer, Turku, Finland). Prior risk values (based on maternal age) were also previously calculated as part of the first trimester screening. Women consented to the use of remnant serum for anonymous scientific purposes according to the Code of Proper Secondary Use of Tissue (2002) issued by the Dutch Federation of Biomedical Scientific Societies. The first trimester screening also included ultrasonographic measurement of fetal nuchal translucency (NT). As the focus of this paper is on evaluation of a biochemical laboratory technique and NT measurement is a different kind of technique, subject to, for example, interoperator variation, NT data were not used in this study.

### 2.2. Array Development

Analytes measured on the array were selected based on studies in our institute as well as the literature. Corresponding antibodies were initially selected based on commercial availability for ELISA purposes. For development of the Ab-array, antibody combinations per marker were first tested on an array as individual (single-plex) immunoassays, using dilutions of a reference sample. This reference sample was a mix composed of serum samples from the DS screening serum bank. This single-plex step comprised optimization of concentrations for capture as well as detection antibodies, determining a suitable serum dilution, and ensuring that in the serum dilution range the signal showed a linear agreement with the amount of serum input used. Next, we tested the antibody combinations for the absence of detectable cross-reactivity. For this, the reference serum was incubated on a multiplex Ab-array (using methods described below), followed by a detection step using various combinations of detection antibodies, to ensure the signal for each analyte was not affected in the multiplex Ab-array.

### 2.3. Antibodies and Standards

We used the following monoclonal and polyclonal capture and detection antibodies: PAPP-A (capture, Hytest, Turku, Finland; detection, R&D Systems, Minneapolis, MN, USA), f*β*-hCG (capture, Acris Antibodies GmbH, Herford, Germany; detection, Hytest), AFP (both from Hytest), ANGPTL3 (both from R&D Systems), EGF (both from R&D Systems), IGFII (capture, Abcam, Cambridge, UK; detection, R&D Systems), SOD1 (capture, R&D Systems; detection, Hytest), and IgG (H + L) (Invitrogen, Breda, The Netherlands). Note that IgG was added for quality control purposes. Standards for PAPP-A and f*β*-hCG were obtained from the AutoDELFIA kits. These standards were calibrated against the WHO International Reference Preparation (PAPP-A: 78/610 for SP1, f*β*-hCG: 75/551). In addition to serum samples from the serum bank, the reference serum was used to correct for interarray variation.

### 2.4. Antibody Arrays and Immunoassays

Capture antibodies were diluted in 2x Protein Arraying Buffer (Maine Manufacturing, Sanford, ME, USA) to a concentration of 0.5–1 mg/mL. Antibodies were spotted on 64-array ONCYTE Avid nitrocellulose film-slides (GRACE Bio-Labs, Bend, Oregon) using a Piezorray Noncontact Microarraying System (PerkinElmer, Wellesley, MA, USA). Two drops per position, of an estimated 330 pL/drop, were spotted under humidity below 40%. Three replicates of each antibody were arrayed to ensure adequate statistics. The slides were stored in a desiccator cabinet (Nalgene, Rochester, NY, USA). Each antibody array slide contained 64 array pads. Of these, 48 were used for analysis of serum samples, 14 were used for control standards (7 for PAPP-A and 7 for f*β*-hCG), and 2 for the reference serum. Small serum volumes (5–25 *μ*L) were diluted ranging from 1 : 5 to 1 : 20.

Arrays were blocked at room temperature (RT) for 1 hour (h) with 70 *μ*L 1x Protein Array Blocking Buffer (Maine Manufacturing), then washed six times for 2.5 min with 70 *μ*L 1x Protein Array Washing Buffer (Maine Manufacturing) using an automatic washing station, and incubated with 70 *μ*L diluted serum or pooled PAPP-A and f*β*-hCG standards, at RT for 1 h. Next, arrays were washed six times for 2.5 min with 70 *μ*L 1x Washing Buffer (WB) (PBS pH 7.4 with 0.05% Tween-20, Sigma). This was followed by incubation with 70 *μ*L of diluted biotinylated detection antibodies, supplemented with 0.1% bovine serum albumin Fraction V (Roche Diagnostics, Mannheim, Germany) and 2% heat-inactivated goat serum (Jackson ImmunoResearch Laboratories, West Grove, PA, USA), at RT for 1 h. Slides were washed (six times for 2.5 min with 70 *μ*L 1x WB) and incubated with 70 *μ*L Streptavidin-Alexa Fluor-647 (Jackson ImmunoResearch Laboratories, diluted 500x in WB) at RT for 30 min. Finally, slides were washed six times for 2.5 min with 70 *μ*L 1x WB and twice with 70 *μ*L deionised water and dried by vacuum.

### 2.5. Data Extraction

Slides were scanned with a Confocal Microarray Scanner (PerkinElmer) at 10 *μ*m resolution. ScanArray Express software V4.0 (PerkinElmer) was used to quantify spot intensity using the adaptive circle method. Corrected median intensity values for each spot (median intensity minus local median background) were used for further analysis. Median intensity values of the three replicate spots were calculated using Microsoft Excel. For the reference serum, the median intensity values for two array pads on a slide were averaged for further analysis. After completion of all assays and quality control (based on inspection of the scan image, replicate consistency, and the signal/background ratios), data for 669 samples (170 cases and 499 controls, always at least two controls per case) were available for further processing and statistical analysis. These steps were performed in R (http://www.r-project.org/) and Microsoft Excel. For PAPP-A and f*β*-hCG, serum concentrations were calculated from array signal intensity using a calibration curve fitted to signal intensities obtained with PAPP-A and f*β*-hCG AutoDELFIA protein standards. Parameter fitting was performed in R based on the parameter logistic log (4PL) model *Y*(*x*) = *D* + ((*A* − *D*)/(1 + (*x*/*C*)^*B*^)) [7].

### 2.6. Statistical Analysis

For all analytes, relative serum levels were calculated by comparing the array signal for an analyte in a sample to that for the reference serum on a different pad on the same slide and multiplying the analyte value by the ratio of the average reference value across all arrays to the average reference value on the same array. This linear scaling correction served to remove variation due to experimental factors in array processing. This scaling calculation was also performed for PAPP-A and f*β*-hCG to allow comparisons of this calculation to the 4PL model. Next, serum concentrations for the various analytes were converted into a multiple of the gestational median value (MoM) following the method described by Cuckle and Wald [8]. For all analytes, the ratio between the geometric average MoMs of DS-cases and controls were calculated. Student's *t*-tests were performed (on log-transformed data) to calculate whether fold ratios were statistically significant. These calculations were also performed for 10, 11, and 12 weeks separately.

The value of serum analytes for a DS prediction model was further tested using logistic regression algorithms and fivefold cross-validation in R statistical software. All serum samples were divided into five groups by stratified randomization to give equal distribution of gestational ages across groups. Logistic regression models were fitted using prior risks and log-MoM data for one or multiple analytes, from 4 out of 5 groups. These models were then used to predict the remaining group using data for prior risk and the corresponding analytes. Model predictive performances were evaluated on the overall data set. The obtained prediction scores were used to calculate the overall Detection Rate (DR, sensitivity) at fixed 5% False Positive Rate (FPR, 1-specificity) as well as the Area under the Curve (AUC) in a Receiver Operating Characteristic (ROC) plot. Markers were considered as having predictive value if they improved the DR as well as AUC.

## 3. Results

### 3.1. Array Development

To test the signal consistency of our multimarker Ab-array, antibody pairs for each analyte were tested both individually and in combinations. No cross-hybridization was observed. Using a reference serum, we found coefficient of variation values per analyte ranging from 8% (f*β*-hCG) to 17% (IgG), with a median value of 11%.

### 3.2. Methods for Marker Measurement

**Figure 1:**
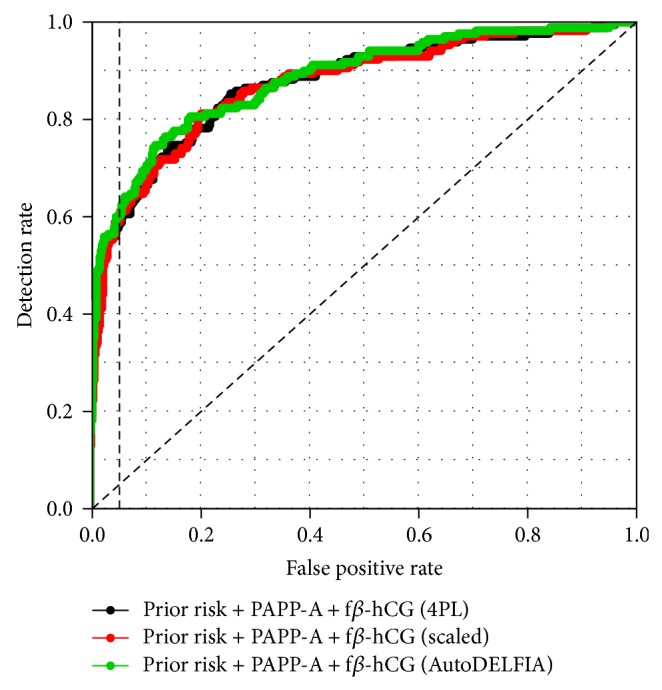
Receiver operating characteristic curves (ROCs) for Down Syndrome prediction models using different measurement methods for PAPP-A and f*β*-hCG.

PAPP-A and f*β*-hCG serum concentrations obtained using Ab-arrays were compared with previously obtained AutoDELFIA data. This comparison was made for Ab-array values based on a 4PL calibration curve model as well as linear scaling compared to a reference standard. Correlations between all three methods are consistently high for both markers (Table 1). This also applies to the log-MoMmed values that serve for prediction modelling (Table 1), and accordingly the ROC curves based on PAPP-A and f*β*-hCG (Figure 1). This confirms the validity of our Ab-array measurement compared to AutoDELFIA. Moreover, it indicates scaling is a suitable alternative for a 4PL-model.

### 3.3. Marker Analysis

Statistical comparison between DS and control pregnancies in our cohort, using the log-MoMmed serum concentrations, shows significant differences for PAPP-A and f*β*-hCG (Table 2). This applies to the values obtained using 4PL calibration and scaling, as well as previously obtained AutoDELFIA data. When the results for individual weeks are compared, the effect magnitude for PAPP-A appears more pronounced at week 10, whereas the effect size and associated *p* value for f*β*-hCG are stronger at week 12. This finding is found across the three measurement methods used (Table 2). It should be noted that the number of samples before week 10 (*n* = 7) or after week 12 (*n* = 18) was not sufficient to allow meaningful calculations on these subsets.

For the other Ab-array markers, concentration differences between cases and controls were less prominent. AFP concentrations showed a significant decrease, but only at week 12 (*p* = 0.017) (Table 2). Also, IGFII concentrations showed a significant increase for the complete cohort (*p* = 0.0098), but not for any of the subgroups (Table 2). The other markers (ANGPTL3, EGF, and SOD1) and the quality control (IgG) showed no significant difference in concentration.

### 3.4. Risk Prediction Modelling

Prediction models based on prior risk, PAPP-A and f*β*-hCG, gave comparable results for the three measurement methods (Table 3). This corroborates our findings that our Ab-array data have comparable reliability as those obtained using AutoDELFIA, as well as that scaling is a practical alternative to using a 4PL calibration curve.

Other markers were tested by determining their added value on two models: firstly to a model using only prior risk and additionally to a model using prior risk and scaled data for PAPP-A and f*β*-hCG. We opted to use scaled data for PAPP-A and f*β*-hCG in this comparison as the data used for the other markers was also obtained using scaling, thus allowing a consistent workflow for all data. Of the markers tested, AFP and IGFII improved the DR and AUC with a very small difference (1% DR, <1% in AUC) when added to the prior risk model. When the comparison was made against the model based on prior risk, PAPP-A and f*β*-hCG, AFP and EGF both increased the DR by 1% but did not improve the AUC.

## 4. Discussion

Antibody arrays are a type of immunoassay that allow for the high-throughput measurement of multiple markers in small sample volumes. These properties make them of interest for population screening programs, including prenatal screening. Combining multiple markers would allow for higher sensitivity as well as specificity for pregnancy outcomes such as DS, other fetal aneuploidies, PE, and IUGR. Additionally, a multimarker array can combine different screening assays, such as those mentioned above, into a single first trimester screening test. This can lead to improved throughput at lower costs, which would be especially advantageous for fetal and maternal health care in low or middle income countries.

Previous studies at our institute have shown that Ab-arrays can be used to quantitatively measure the current DS screening serum markers (PAPP-A and f*β*-hCG) within one assay using small serum volumes [6]. However, to take this methodology beyond proof-of-principle studies, larger studies are necessary. Larger studies would allow for a more meaningful comparison of serum measurements obtained by Ab-arrays versus current screening practice. Moreover, larger studies allow including subsequent data analysis steps in the evaluation, such as correcting marker levels for gestational age and prediction modelling. For implementation in a screening setting, the predictive performance of an Ab-array should at least match that of current screening methodology or improve upon it by including additional markers. In this study, we performed such an evaluation of our Ab-array based on PAPP-A and f*β*-hCG, as well as other candidate markers.

The results for PAPP-A and f*β*-hCG are very encouraging. Not only were serum measurements on the Ab-array highly correlated with those obtained by AutoDELFIA (Table 1), but also this applied to the DS prediction modelling results (Table 3, Figure 1). Levels of PAPP-A were more affected at week 10, and those of f*β*-hCG were more affected at week 12. This is consistent with literature findings that predictive performance for PAPP-A is the highest at week 10 or earlier in the first trimester, whereas that of f*β*-hCG is the highest several weeks later [9, 10].

To further assess the possibilities for multiplexed serum screening, we expanded our Ab-array with a set of prioritized candidate biomarkers for prenatal screening endpoints, based on previous studies in our institute as well as the literature. This resulted in five additional candidate biomarkers being added to the Ab-array. For these markers we did not use calibration standards due to unavailability or space limitations. Given the high correlations between signal calculation methods for PAPP-A and f*β*-hCG, and a linear signal response observed in the array development phase, we assume that for these markers scaling-based data are a reliably alternative.

The first of these five additional markers is AFP. This is a well-known second trimester DS screening marker, although AFP is also predictive in the first trimester [11, 12]. We also found significantly decreased levels of AFP in a multimarker serum screening study [2], although this finding was not confirmed in the subsequent validation study. In this study, AFP serum levels were not significantly affected for the whole cohort, although there was a trend from a significant decrease in serum of 12 weeks GA, via a nonsignificant decrease in week 11, to no change in week 10 (Table 2). This is in line with literature reports that AFP—best known as a second trimester biomarker—can also distinguish between DS and unaffected pregnancies in the first trimester, but that its discriminating power increases with gestational age [9, 12]. In prediction risk modelling, AFP only allowed for a small increase in DR when added to the prior risk model, as well as to the model based on prior risk, PAPP-A and f*β*-hCG. Taken together, although the effects per week are in agreement with the literature on AFP as biomarker, the 1% increase in DR on the overall cohort is too small to be of practical use for adding predictive power in future implementation.

EGF was added as a second prioritized candidate marker. EGF inhibits apoptosis of trophoblasts and promotes their differentiation and invasion [13–15]. This EGF-induced differentiation results in secretion of human chorionic gonadotropin [13]. As this latter marker is affected in DS pregnancies, the potential value of EGF in DS screening is conceivable. Moreover, reduced EGF levels have been reported for preeclampsia [16] as well as IUGR [17]. We previously reported decreased EGF levels in our initial serum screening study [2] as well as in the subsequent validation study, using serum of 12-week GA. Unfortunately, we were not able to corroborate our previous EGF findings with our Ab-array. EGF did not show a significant difference in serum levels, nor did it help to improve the prediction modelling (Tables 2 and 3). This is in line with the study of Mastricci et al., who found no significant difference in EGF serum concentration in a cohort of 11 + 0 to 13 + 6 weeks' GA [18]. Interestingly, our data show decreased levels of EGF at 12 weeks' GA, whereas EGF levels were increased at weeks 10 and 11. This might indicate that, as for AFP, the predictive power of EGF depends on the gestational age. Nevertheless, this study, combined with that by Mastricci et al., indicates that EGF is probably not useful for improving DS serum screening.

We also added two markers which we identified as novel potential markers by data mining [3, 5], namely, ANGPTL3 (for DS) and IGFII (for both DS and PE). Of these markers, IGFII showed a significant increase for DS pregnancies in the overall cohort (Table 2), and a small improvement to the prior risk model (Table 3). This indicates that IGFII might be considered as a novel DS screening marker and as such supports our data mining approach. However, as IGFII does not help improve the current serum screening model (prior risk, PAPP-A, and f*β*-hCG) the added value of IGFII for a large-scale screening program setting is probably limited.

Finally, we included SOD1 on our Ab-array based on several kinds of evidence. SOD1 represents one of the best studied genes and proteins in relation to DS. The number of literature studies on SOD1 in relation to DS is comparable to that of established screening markers. A major reason for this is that SOD1 is located on human chromosome 21 in a region traditionally referred to as the “Down Syndrome Critical Region,” although nowadays this particular term is less accepted [19]. In a previous study by our groups, SOD1 showed increased gene expression in both placenta and fetal liver of the Ts1Cje mouse model for DS [4]. Changes in human serum SOD activity levels have also been reported for preeclampsia and the corresponding change in antioxidant activity has been associated with PE pathogenesis [20, 21]. Whereas increased SOD1 protein levels have been found in human placentas of DS pregnancies [22] and higher numbers of SOD1 DNA fragments have been described in maternal circulation of DS pregnancies [23], to our knowledge the use of SOD1 protein as a serum markers for DS screening has not yet been investigated. In our data set, the SOD1 serum protein level showed no significant difference, nor did it help improve the risk prediction modelling (Tables 2 and 3). This indicates that SOD1 is probably not of interest as a serum protein biomarker for DS pregnancies.

## 5. Conclusion

Our results show that Ab-array methodology is appropriate for large-scale quantitative PAPP-A and f*β*-hCG measurements. We also show for the first time that for application in DS prediction, multiplex Ab-arrays perform as accurately as current immunoassay methods. Including additional markers on the Ab-array did not help to further improve DS predictive performance beyond current standards. Further implementation of Ab-arrays in a prenatal screening setting for fetal and maternal health will require additional other first trimester serum screening markers.

## Figures and Tables

**Table 1:** Correlations (*R*) between measurement methods for PAPP-A and f*β*-hCG. All *R*-values have corresponding *p* values < 10^−8^.

	Serum concentrations PAPP-A			Log-MoM values PAPP-A		
	Array, 4PL	Array, scaled	AutoDELFIA	Array, 4PL	Array, scaled	AutoDELFIA
Array, 4PL	1	0.98	0.90	1	0.97	0.82
Array, scaled	0.98	1	0.88	0.97	1	0.79
AutoDELFIA	0.90	0.88	1	0.82	0.79	1

	Serum concentrations f*β*-hCG			Log-MoM values f*β*-hCG		
	Array, 4PL	Array, scaled	AutoDELFIA	Array, 4PL	Array, scaled	AutoDELFIA

Array, 4PL	1	0.99	0.95	1	0.99	0.92
Array, scaled	0.99	1	0.95	0.99	1	0.93
AutoDELFIA	0.95	0.95	1	0.92	0.93	1

**Table 2:** Ratio of MoM-adjusted serum concentrations per analyte for DS compared to controls. Significance is indicated as ^∗^ *p* < 0.05; ^∗∗^ *p* < 0.01; ^∗∗∗^ *p* < 0.001.

	Total cohort (nDS = 170)	Week 10 (nDS = 43)	Week 11 (nDS = 25)	Week 12 (nDS = 77)
PAPP-A (4PL)	0.52^∗∗∗^	0.41^∗∗∗^	0.52^∗∗∗^	0.60^∗∗∗^
PAPP-A (scaled)	0.56^∗∗∗^	0.46^∗∗∗^	0.55^∗∗∗^	0.65^∗∗∗^
PAPP-A (AutoDELFIA)	0.43^∗∗∗^	0.30^∗∗∗^	0.42^∗∗∗^	0.56^∗∗∗^
f*β*-hCG (4PL)	1.74^∗∗∗^	1.49^∗^	1.98^∗∗∗^	1.81^∗∗∗^
f*β*-hCG (scaled)	1.99^∗∗∗^	1.55^∗^	2.38^∗∗∗^	2.11^∗∗∗^
f*β*-hCG (autoDELFIA)	1.57^∗∗∗^	1.25	1.71^∗∗∗^	1.75^∗∗∗^
AFP	0.90	1.01	0.85	0.83^∗^
ANGPTL3	0.98	1.07	0.96	0.92
EGF	1.12	1.17	1.39	0.93
IGFII	1.32^∗∗^	1.40	1.47	1.17
SOD1	1.04	1.11	1.14	0.93
IgG	1.13	1.32	1.04	1.04

**Table 3:** Model predicted DS detection rate (and 95% CI) for 5% False Positive Rate and corresponding Area under the Curve, for models based on prior risk and several marker combinations. DR, detection rate; CI, confidence interval; AUC, Area under the Curve.

Model	DR (%)	DR 95% CI	AUC
Prior risk	27	(19–36)	0.741
Prior risk + PAPP-A + f*β*-hCG (4PL)	58	(49–66)	0.873
Prior risk + PAPP-A + f*β*-hCG (scaled)	58	(49–67)	0.870
Prior risk + PAPP-A + f*β*-hCG (AutoDelfia)	59	(51–68)	0.880
Prior risk + AFP	28	(19–39)	0.748
Prior risk + PAPP-A + f*β*-hCG (scaled) + AFP	59	(49–68)	0.869
Prior risk + ANGPTL3	26	(16–36)	0.732
Prior risk + PAPP-A + f*β*-hCG (scaled) + ANGPTL3	58	(48–66)	0.869
Prior risk + EGF	25	(17–34)	0.739
Prior risk + PAPP-A + f*β*-hCG (scaled) + EGF	59	(50–67)	0.870
Prior risk + IGF2	28	(18–38)	0.742
Prior risk + PAPP-A + f*β*-hCG (scaled) + IGF2	58	(49–66)	0.870
Prior risk + SOD1	19	(10–31)	0.736
Prior risk + PAPP-A + f*β*-hCG (scaled) + SOD1	58	(49–66)	0.870
Prior risk + IgG	19	(9–31)	0.739
Prior risk + PAPP-A + f*β*-hCG (scaled) + IgG	57	(49–66)	0.872
